# An association study of *IL2RA* polymorphisms with cerebral palsy in a Chinese population

**DOI:** 10.1186/s12920-022-01350-5

**Published:** 2022-10-04

**Authors:** Yimeng Qiao, Yangong Wang, Yiran Xu, Jin Zhang, Yu Su, Ye Cheng, Dan Bi, Juan Song, Lei Xia, Ming Li, Xiaoli Zhang, Dengna Zhu, Ting Wang, Jian Ding, Xiaoyang Wang, Changlian Zhu, Qinghe Xing

**Affiliations:** 1 Children’s hospital of Fudan University; and Institutes of Biomedical Sciences of Fudan University, Wanyuan Road 399, Shanghai, 201102 China; 2 Henan Key Laboratory of Child Brain Injury, Department of Pediatrics, the 3Rd Affiliated Hospital of Zhengzhou University and Institute of Neuroscience, Kangfuqian Street 7, Zhengzhou, 450052 China; 3 Henan Key Laboratory of Child Brain Injury and Henan Pediatric Clinical Research Center, Department of Pediatrics, the 3Rd Affiliated Hospital and Institute of Neuroscience of Zhengzhou University, Zhengzhou, 450052 China; 4 Henan Institute of Reproduction Health Science and Technology, No.26 Jingwu Road, Jinshui District, Zhengzhou, 450002 Henan China; 5 Centre of Perinatal Medicine and Health, Sahlgrenska Academy, University of Gothenburg, 40530 Gothenburg, Sweden; 6 Department of Women’s and Children’s Health, Karolinska Institutet, 17177 Stockholm, Sweden; 7 Center for Brain Repair and Rehabilitation, Institute of Neuroscience and Physiology, Sahlgrenska Academy, University of Gothenburg, 40530 Gothenburg, Sweden; 8 Shanghai Center for Women and Children’s Health, Shanghai, 200062 China

**Keywords:** Association study, *IL2RA*, Cerebral palsy, Chinese population, Gene polymorphism

## Abstract

**Background:**

Cerebral palsy (CP), the most common physical disability of childhood, is a nonprogressive movement disorder syndrome. Eighty percent of cases are considered idiopathic without a clear cause. Evidence has shown that cytokine abnormalities are widely thought to contribute to CP.

**Methods:**

An association between 6 SNPs (rs12244380, rs2025345, rs12722561, rs4749926, rs2104286 and rs706778) in *IL2RA* (interleukin 2 receptor subunit alpha) and CP was investigated using a case–control method based on 782 CP cases and 778 controls. The allele, genotype and haplotype frequencies of SNPs were assessed using the SHEsis program. Subgroup analyses based on complications and clinical subtypes were also conducted.

**Results:**

Globally, no differences in genotype or allele frequencies for any SNPs remained significant after Bonferroni correction between patients and controls, except rs706778, which deviated from Hardy–Weinberg equilibrium and was excluded from further analyses. However, subgroup analysis revealed a significant association of rs2025345 with spastic tetraplegia (P genotype = 0.048 after correction) and rs12722561 with CP accompanied by global developmental delay (P allele = 0.045 after correction), even after Bonferroni correction.

**Conclusions:**

These findings indicated that genetic variations in *IL2RA* are significantly associated with CP susceptibility in the Chinese Han population, suggesting that *IL2RA* is likely involved in the pathogenesis of CP. Further investigation with a larger sample size in a multiethnic population is needed to confirm the association.

## Background

Cerebral palsy (CP) is a nonprogressive movement disorder caused by brain damage occurring in the developing fetus or infant [1, 2]. It is a group of permanent disorders that can affect the development of sports and lead to restricted activities [3]. CP is one of the most common physical disability diseases in children, with an incidence of 1.5–2.5/1,000 live births [4]. Although some studies have pointed out that risk factors, such as intrauterine infection, hypoxic-ischemia insults, and central nervous system infections, can result in CP [5, 6], 80% of cases are considered idiopathic without a clear cause [7]. It has been reported that CP children are prone to have congenital anomalies, the incidence is higher in identical twins than in fraternal twins, and the risk of CP is higher in consanguineous families than nonconsanguineous families [8], suggesting that genetic factors may play an important role in the etiology of CP. Moreover, genes such as *NOS1*, *OLIG2*, *ATG5*, *ATG7* and *IL-6* have been significantly associated with susceptibility to CP [9–12].

CP is linked to immune dysfunction, including altered serum and cerebrospinal fluid cytokine profiles and aberrant cell-mediated immune responses [13]. Inflammation is one etiologic component of brain white matter damage acquired during early development that gives rise to CP [14, 15]. In addition, cytokines, such as interleukins and interferons, are a group of small and secreted proteins that can mediate normal and continuous signal transduction between nonimmune tissue cells, including the central nervous system (CNS) [16]. Cytokines are of particular importance during neural development and function at all stages and are associated with CP and other neurodevelopmental disorders (NDDs). Magalhaes et al. analyzed the relationship between inflammatory molecules and neurodevelopment and found that higher circulating levels of *IL-1β*, *IL-6*, *TNF* and *CXCL8/IL-8* were associated with neurological abnormalities in CP children [17]. Furthermore, studies have reported that *IL-23R*, *IL-8* and *IL-6* gene polymorphisms are related to CP in the Chinese population [18–20].

Interleukin-2 (IL-2), the first cytokine to be molecularly cloned, is a typical alpha helix cytokine that binds to and transmits signals through the IL-2 receptor (IL2R) complex, which consists of 3 different subunits, IL2RA (interleukin 2 receptor subunit alpha), IL2RB (interleukin 2 receptor subunit beta), and IL2RG (interleukin 2 receptor subunit gamma) [21, 22]. Previous studies have proven that the IL-2/IL2R complex signaling pathway plays a key role in the proliferation of T cells and the generation of effector and memory cells to promote immune responses. Furthermore, the IL-2/IL2R complex signaling pathway can promote the generation, survival, and functional activity of Treg cells to control immune responses and maintain self-tolerance [23]. Damaging mutations disrupting this pathway can cause severe forms of Mendelian immune dysregulation [22, 23]. IL2RA forms the largest of the three subunit interfaces, which, together with the high abundance of charge-charge interactions, correlates well with the rapid association rate and high-affinity interaction of IL2RA with IL-2 at the cell surface [24, 25]. IL2RA deficiency can result in human immune-mediated diseases [26–28].

Based on the above findings, we speculate that *IL2RA* may be associated with susceptibility to CP. However, it remains unknown whether *IL2RA* is associated with CP. Therefore, we used a case–control study to explore the possible association of *IL2RA* with CP, which will provide genetic evidence for the role of *IL2RA* in the etiology of CP and its related potential mechanisms.

## Methods

### Participants

In our study, 782 children with CP and 778 healthy controls were recruited from the centers for CP rehabilitation and Child Health Care Departments in the Third Affiliated Hospital of Zhengzhou University, Zhengzhou Children’s Hospital. This study was approved by the Ethics Committee of Zhengzhou University (No: 2017–09) in accordance with the principles of the Declaration of Helsinki. Statements of informed consent were obtained from the guardians of all children after full explanation of the procedure. The case group consisted of 542 males (69.3%) and 240 females (30.7%), and the mean age was 18.5 ± 15.4 months. The control group consisted of 778 healthy children, including 520 males (66.8%) and 258 females (33.2%), and the mean age was 19.3 ± 16.8 months (Table 1).

**Table 1:** Clinical characteristics of all participants

Characteristic	CP cases (n = 782)	Controls (n = 778)
Sex (male: female)	542:240	520:258
Preterm (< 37 weeks)	47	10
Low Birth Weight (< 2500 g)	40	2
Birth Asphyxia	229	13
Type of CP		
Spastic CP	522	NA
CP with quadriplegia	284	NA
CP with diplegia	126	NA
Complications		
CP with NE	310	NA
CP with GDD	299	NA
Maternal factors		
PIH	26	NA

*CP* cerebral palsy, *GDD* Global developmental delay, *NE* neonatal encephalopathy, *PIH* pregnancy-induced hypertension

### CP diagnosis, classification and exclusion criteria

In the case group, children diagnosed with congenital metabolic diseases and myopathy as well as children with a family history of nervous system diseases were excluded. Pediatric rehabilitation specialists ensured CP diagnosis using standard criteria related to nonprogressive disorders of movement control and posture [1]. Every participant received a detailed clinical evaluation with comprehensive pretest counseling.

Clinical information, including demographic variables (such as sex, gestational age, mode of delivery, singletons or twins), known risk factors (such as pregnancy-induced hypertension (PIH), perinatal asphyxia, threatened premature labor), CP complications (such as global developmental delay, intellectual disability) and neonatal complications, were all recorded if available.

Quadriplegia, one subtype of CP, is classified by the number and distribution of the impaired limbs [29, 30]. Quadriplegia was diagnosed when both the upper and lower limbs of the patients were paralyzed. Global developmental delay (GDD) diagnosis was limited to individuals under five years old when they were significant delay in at least two developmental domains: including motor skills, speech and language, cognitive skills, and social and emotional skills [31].

### Genotyping and statistical analysis

**Fig. 1:**
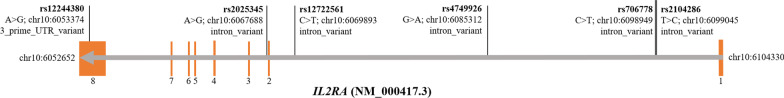
The location of studied SNPs in *IL2RA* (NM_000417.3, chr10:6,052,652-6,104,330). Yellow boxes represent exons of *IL2RA*

Peripheral blood samples were obtained from the participants for genomic DNA extraction. Altogether, 6 SNPs (rs12244380, rs2025345, rs12722561, rs4749926, rs2104286 and rs706778) in *IL2RA* gene with minor allele frequency (MAF) in the Chinese Han population greater than 0.1 were selected from the dbSNP database (https://www.ncbi.nlm.nih.gov/snp/) and the phase II genotyping data of the HapMap project (http://www.1000genomes.org/). All 6 SNPs were reported in published literature online, which were associated with immune dysfunction, such as type 1 diabetes, autoimmune disease. Rs12244380 (exon 8, chr10:6,053,374) is located in 3’UTR, while the other 5 SNPs are located in introns: rs2025345 (intron 2, chr10:6,067,688), rs12722561 (intron 1, chr10:6,069,893), rs4749926 (intron 1, chr10:6,085,312), rs2104286 (intron 1, chr10:6,099,045) and rs706778 (intron 1, chr10:6,098,949) (Fig. 1). According to the single nucleotide polymorphism (SNP) location in *IL2RA*, a MAF > 0.1 and potential function, six SNPs were selected as candidates and genotyped by the MassARRAY system (Shanghai Perchant Biotechnology Co., Ltd. synthesized primers and probes).

Statistical analysis was performed with SHEsis, an online program (http://analysis.bio-x.cn/) that can test Hardy–Weinberg equilibrium (HWE) and linkage disequilibrium (LD) and calculate allele frequencies, genotype frequencies and haplotype frequencies in the case and control groups. The P values were two-tailed, and *P* < 0.05 was considered significant. The odds ratio (OR) and its 95% confidence interval (CI) were also calculated. The Bonferroni correction was applied to account for multiple testing on each individual SNP and haplotype. G*power 3.1 software was used to evaluate statistical efficacy.

## Results

### Overall analysis

Power calculations analysis showed that the sample size in this study had > 85% power to detect a significant association with an effect size index of 0.1 (α < 0.05). Except for rs706778 (*P* = 0.042), the genotype distribution of the selected SNPs did not deviate from HWE in the control population (*P* > 0.05). Therefore, rs706778 was excluded from further testing. For the other 5 SNPs, rs12244380, rs2025345, rs12722561, rs4749926 and rs2104286, the allele frequency of rs12722561 (*P* = 0.025) was different between all CP patients and the control group, but the difference disappeared after Bonferroni correction. There were no significant differences in the allele or genotype frequencies of rs12244380, rs2025345, rs4749926 and rs2104286 between CP cases and controls (Table 2).

**Table 2:** Genotype and allele frequencies of SNPs in *IL2RA* between CP and controls

Group	Allele frequency		*P* value	OR [95% CI]	Genotype frequency			*P* value
rs12244380	A	G			A/A	A/G	G/G	
CP	1243 (0.874)	179 (0.126)	0.401	1.098 [0.883–1.364]	543 (0.764)	157 (0.221)	11 (0.015)	0.628
Control	1240 (0.864)	196 (0.136)			533 (0.742)	174 (0.242)	11 (0.015)	
rs2025345	A	G	0.341	0.929 [0.798–1.081]	A/A	A/G	G/G	0.227
CP	907 (0.627)	539 (0.373)			275 (0.380)	357 (0.494)	91 (0.126)	
Control	915 (0.644)	505 (0.356)			298 (0.420)	319 (0.449)	93 (0.131)	
rs12722561	C	T	0.025^a^	1.284 [1.031–1.599]	C/C	C/T	T/T	0.080
CP	1307 (0.887)	167 (0.113)			579 (0.786)	149 (0.202)	9 (0.012)	
Control	1225 (0.859)	201 (0.141)			525 (0.736)	175 (0.245)	13 (0.018)	
rs4749926	A	G	0.981	1.002 [0.865–1.160]	A/A	A/G	G/G	0.983
CP	595 (0.395)	911 (0.605)			105 (0.139)	385 (0.511)	263 (0.349)	
Control	592 (0.395)	908 (0.605)			106 (0.141)	380 (0.507)	264 (0.352)	
rs2104286	C	T	0.127	0.844 [0.679–1.050]	C/C	C/T	T/T	0.166
CP	172 (0.116)	1312 (0.884)			3 (0.004)	166 (0.224)	573 (0.772)	
Control	200 (0.134)	1288 (0.866)			8 (0.011)	184 (0.247)	552 (0.742)	

*P* value after Bonfferoni: a: 0.126

*OR* odds ratio, *CI* confidence interval

Haplotype analysis is a powerful strategy to determine whether SNPs have greater predictive value when analyzed together. These polymorphisms, rs2025345, rs12722561 and rs4749926, exhibit a strong LD (D’ > 0.85) and form four principal haplotypes (ACA, ACG, ATG and GCA). The ATG haplotype was associated with CP (*P* = 0.026), but the difference disappeared after correction (Table 3).

**Table 3:** Haplotype analysis of rs2025345-rs12722561-rs4749926

Haplotype	Case (frequency)	Control (frequency)	p-value	OR [95% CI]
ACA	19.77 (0.038)	68.12 (0.050)	0.231	0.732 [0.439–1.221]
ACG	253.28 (0.483)	619.74 (0.456)	0.410	1.090 [0.888–1.336]
ATG	48.32 (0.092)	173.26 (0.128)	0.026	0.684 [0.488–0.957]
GCA	191.58 (0.366)	450.93 (0.332)	0.231	1.138 [0.921–1.407]

### Subgroup analysis

CP is a syndrome with strong phenotypic and etiological heterogeneity. The genetic diagnosis rate of different clinical subtypes of CP is inconsistent, suggesting that different types of CP may have different genetic etiologies. Then, we conducted a subgroup analysis to explore the association of different CP types with *IL2RA* genetic variants. The results indicated that the association of spastic tetraplegia with rs2025345 (OR = 0.868, 95% CI = 0.706–1.066, P genotype = 0.0097) and the association of CP + GDD with rs12722561 (OR = 1.518, 95% CI = 1.107–2.082, P allele = 0.0091) were both significant, even after Bonferroni correction. Moreover, the analysis of the other three SNPs in CP types and controls revealed a nonsignificant difference in allelic and genotypic distributions (Tables 4 and 5).

**Table 4:** Allele and genotype frequencies of *IL2RA* in CP with spastic tetraplegia and controls

Group	Allele frequency		*P* value	OR [95% CI]	Genotype frequency			*P* value
rs12244380	A	G			A/A	A/G	G/G	
CP	456 (0.857)	76 (0.143)	0.716	0.948 [0.713–1.262]	198 (0.744)	60 (0.226)	8 (0.030)	0.298
Control	1240 (0.864)	196 (0.136)			533 (0.742)	174 (0.242)	11 (0.015)	
rs2025345	A	G	0.177	0.868 [0.707–1.066]	A/A	A/G	G/G	0.0097^a^
CP	324 (0.611)	206 (0.389)			88 (0.332)	148 (0.558)	29 (0.109)	
Control	915 (0.644)	505 (0.356)			298 (0.420)	319 (0.449)	93 (0.131)	
rs12722561	C	T	0.053	1.354 [0.995–1.844]	C/C	C/T	T/T	0.137
CP	487 (0.892)	59 (0.108)			216 (0.791)	55 (0.201)	2 (0.007)	
Control	1225 (0.859)	201 (0.141)			525 (0.736)	175 (0.245)	13 (0.018)	
rs4749926	A	G	0.389	1.091 [0.894–1.332]	A/A	A/G	G/G	0.540
CP	227 (0.416)	319 (0.584)			40 (0.147)	147 (0.538)	86 (0.315)	
Control	592 (0.395)	908 (0.605)			106 (0.141)	380 (0.507)	264 (0.352)	
rs2104286	C	T	0.633	0.931 [0.696–1.247]	C/C	C/T	T/T	0.551
CP	70 (0.126)	484 (0.874)			1 (0.004)	68 (0.245)	208 (0.751)	
Control	200 (0.134)	1288 (0.866)			8 (0.011)	184 (0.247)	552 (0.742)	

*P* value after Bonfferoni a: 0.048

**Table 5:** Allele and genotype frequencies of *IL2RA* in CP with GDD and controls

Group	Allele frequency		*P* value	OR [95% CI]	Genotype frequency			*P* value
rs12244380	A	G			A/A	A/G	G/G	
CP	480 (0.873)	70 (0.127)	0.589	1.084 [0.809–1.452]	208 (0.756)	64 (0.233)	3 (0.011)	0.817
Control	1240 (0.864)	196 (0.136)			533 (0.742)	174 (0.242)	11 (0.015)	
rs2025345	A	G	0.350	0.907 [0.740–1.113]	A/A	A/G	G/G	0.554
CP	342 (0.622)	208 (0.378)			105 (0.382)	132 (0.480)	38 (0.138)	
Control	915 (0.644)	505 (0.356)			298 (0.420)	319 (0.449)	93 (0.131)	
rs12722561	C	T	0.0091^a^	1.519 [1.107–2.082]	C/C	C/T	T/T	0.030^b^
CP	509 (0.902)	55 (0.098)			230 (0.816)	49 (0.174)	3 (0.011)	
Control	1225 (0.859)	201 (0.141)			525 (0.736)	175 (0.245)	13 (0.018)	
rs4749926	A	G	0.858	1.018 [0.836–1.239]	A/A	A/G	G/G	0.978
CP	229 (0.399)	345 (0.601)			41 (0.143)	147 (0.512)	99 (0.345)	
Control	592 (0.395)	908 (0.605)			106 (0.141)	380 (0.507)	264 (0.352)	
rs2104286	C	T	0.138	0.795 [0.587–1.077]	C/C	C/T	T/T	0.258
CP	62 (0.110)	502 (0.890)			1 (0.004)	60 (0.213)	221 (0.784)	
Control	200 (0.134)	1288 (0.866)			8 (0.011)	184 (0.247)	552 (0.742)	

*P* value after Bonfferoni: a: 0.045; b: 0.15

The analysis of the association between *IL2RA* and various risk factors detected a significant influence on CP by the interactions between genotype distribution in rs12244380 and pregnancy-induced hypertension; even after Bonferroni correction, the association between them was still significant (OR = 0.531, 95% CI = 0.266–1.059, *P* = 0.0005) (Table 6). However, the proportions of pregnancy-induced hypertension exposure in CP cases and controls presented a large difference, which may have weakened the feasibility of the P value. In addition, rs12722561 presented a significant association with CP + asphyxia, CP + neonatal encephalopathy (NE) and CP + intracranial hemorrhage, but the significance disappeared after Bonferroni correction.

**Table 6:** Allele and genotype frequencies of *IL2RA* in CP with pregnancy-induced hypertension and controls

Group	Allele frequency		*P* value	OR [95% CI]	Genotype frequency			*P* value
rs12244380	A	G			A/A	A/G	G/G	
CP	37 (0.771)	11 (0.229)	0.068	0.531 [0.266–1.059]	16 (0.667)	5 (0.208)	3 (0.125)	0.0005^a^
Control	1240 (0.864)	196 (0.136)			533 (0.742)	174 (0.242)	11 (0.015)	
rs2025345	A	G	0.783	0.920 [0.0.508–1.667]	A/A	A/G	G/G	0.418
CP	30 (0.625)	18 (0.375)			8 (0.333)	14 (0.583)	2 (0.083)	
Control	915 (0.644)	505 (0.356)			298 (0.420)	319 (0.449)	93 (0.131)	
rs12722561	C	T	0.087	0.552 [0.277–1.100]	C/C	C/T	T/T	0.066
CP	37 (0.771)	11 (0.229)			15 (0.625)	7 (0.292)	2 (0.083)	
Control	1225 (0.859)	201 (0.141)			525 (0.736)	175 (0.245)	13 (0.018)	
rs4749926	A	G	0.519	1.205 [0.683–2.127]	A/A	A/G	G/G	0.710
CP	22 (0.440)	28 (0.560)			5 (0.200)	12 (0.480)	8 (0.320)	
Control	592 (0.395)	908 (0.605)			106 (0.141)	380 (0.507)	264 (0.352)	
rs2104286	C	T	0.520	1.288 [0.594–2.792]	C/C	C/T	T/T	0.570
CP	8 (0.167)	40 (0.833)			0 (0.000)	8 (0.333)	16 (0.667)	
Control	200 (0.134)	1288 (0.866)			8 (0.011)	184 (0.247)	552 (0.742)	

*P* value after Bonfferoni: a: 0.0026

## Discussion

CP is the most common physical disability of childhood and is a heterogeneous condition resulting from damage to the developing brain [32]. CP has no curative therapy and few disease-modifying interventions [33]. Earlier and accurate diagnosis of CP has become highly desirable because it allows earlier initiation of treatments that may improve long-term outcomes during periods of rapid brain growth and neuroplasticity, which suggests the importance of uncovering the etiology of CP [34]. However, these known CP causes, such as periventricular leukomalacia (PVL), NE, infarct, and premature delivery, account for only a minority of the total cases [18]. The pathogenesis of CP is largely unknown and needs further study.

Cytokines coordinate the host response to infection and mediate normal, ongoing signaling between cells of nonimmune tissues, including the CNS [16]. Cytokines can profoundly impact fetal neurodevelopment in response to maternal infection or prenatal hypoxia [35]. CP has been linked to early life immune activation and inflammation [36, 37], and several cytokines, including *IL-6*, *IL-8*, *IL-10*, *IL-17* and *IL-23R,* have been proven to be related to CP [18, 34, 38, 39]. IL-2 is a pleiotropic cytokine produced after antigen activation. IL-2 can promote CD8^+^ T cell and natural killer cells cytolytic activity, modulate T-cell differentiation programs in response to antigens and control the development and maintenance of Treg cells to mediate immune responses and maintain self-tolerance by the IL-2/IL2R complex signaling pathway [40]. IL2RA provides a higher affinity to the IL2R complex and a more stable state through which to transmit signals. Studies have shown that the expression of the anti-inflammatory cytokine IL-2 is decreased in infants with CP [41, 42], which implies that the IL2/IL2R complex pathway is related to CP. Here, we first investigated the relationship between CP and *IL2RA* by a case–control study in Han Chinese individuals and found that *IL2RA* was associated with an increased risk of CP.

Several kinds of immune-activated cells have been shown to secrete IL-2, including T cells, natural killer cells, dendritic cells (DCs) and mast cells [43]. During immune activation, IL-2 expression increases rapidly. Activated DCs secrete low levels of IL-2 as an early source, thereby stimulating T cell activation [44]. Activated T cells (including CD4^+^ and CD8^+^ T cells) begin to secrete large amounts of IL-2 for their own use and stimulate adjacent IL2R^+^ cells by paracrine signaling [40, 45, 46]. After IL-2 binds to the IL2R complex, signal transduction occurs through three main pathways: (i) Janus kinase (JAK)-signal transducer and activator of transcription (STAT), (ii) the phosphoinositide 3-kinase (PI3K)-AKT-mammalian target of rapamycin (mTOR)-p70 S6 kinase pathway and (iii) mitogen-activated protein kinase (MAPK). Phosphorylated STAT5A and STAT5B then oligomerize to form STAT5 dimers and tetramers before undergoing nuclear translocation, where they bind to key target genes (including *IL2RA*) responsible for cell activation, differentiation, and proliferation, while mTOR and MAPK promote cell growth and survival [22, 40, 45]. It is reasonable to hypothesize that IL2/IL2R complex signaling pathways are disturbed by functional genetic variants, immune cells are unable to proliferate and differentiate normally and the immune system cannot respond optimally to antigen stimulation, causing damage to the developing brain (CP).

Previous studies have shown that disruption of IL2/IL2R complex signaling pathways can lead to adverse cerebral events. In IL-2 knockout (IL-2 KO) mice, brain IL2R complexes are enriched in hippocampal formation, and loss of IL-2 results in cytoarchitectural alterations in the hippocampus and septum. These alterations include decreased cholinergic somata in the medial septum/vertical limb of the diagonal band of Broca (MS/vDB) and decreased distance across the infrapyramidal (IP) granule cell layer (GCL) of the dentate gyrus (DG) [47]. The deletion of IL-2 alters the neuroimmunological status of the mouse hippocampus through dysregulation of cytokines produced by CNS cells [48]. A study in 2015 suggested that complex interactions between *IL2* deficiency in the brain and the immune system may modify brain processes involved in different modalities of learning and memory [49]. In this study, we analyzed the correlation between five SNPs of *IL2RA* and CP and finally ascertained the association of *IL2RA* with CP. The results of our study showed that there might be an association between *IL2RA* and susceptibility to CP, implying that SNPs in the *IL2RA* gene might be involved in the occurrence and development of CP.

Our study has several limitations. First, our study was based on a single gene for susceptibility to CP. Given the genetic heterogeneity and gene–gene interactions related to CP etiology, other candidate genes that are part of the IL2/IL2R complex signaling pathway should also be analyzed. Second, we were unable to examine IL2RA protein expression in the brains of the subjects in the current study. Future studies are encouraged to measure inflammatory cytokine alterations in the brain. Third, although our study demonstrated an association between *IL2RA* and CP, further functional studies are necessary to verify the results.

## Conclusion

We examined the influence of 6 SNPs (rs12244380, rs2025345, rs12722561, rs4749926, rs2104286 and rs706778) in *IL2RA* on susceptibility to CP and identified *IL2RA* as a risk gene for CP. To our knowledge, the role of genetic variation in *IL2RA* in susceptibility to CP has not been investigated before. The full effect of the *IL2RA* gene on CP cannot be revealed by the analysis of a limited number of SNPs. Future studies on a larger number of samples and on different ethnic groups will be required to better understand the contribution of *IL2RA* variants to the risk of CP.

## Data Availability

The authors are unable to share detailed clinical data due to full anonymization of the data is very difficult. But the datasets analyzed and generated during the study are available from the corresponding author on reasonable request.

## Abbreviations

CP: Cerebral palsy
SNP: Single nucleotide polymorphism
CNS: Central nervous system
OR: Odds ratio
CI: Confidence interval
HWE: Hardy-weinberg equilibrium
MAF: Minor allele frequency
GDD: Global developmental delay
NE: Neonatal encephalopathy
PIH: Pregnancy-induced hypertension
